# Decision Making in Mice During an Optimized Touchscreen Spatial Working Memory Task Sensitive to Medial Prefrontal Cortex Inactivation and NMDA Receptor Hypofunction

**DOI:** 10.3389/fnins.2022.905736

**Published:** 2022-05-17

**Authors:** Tyler D. Dexter, Daniel Palmer, Ahmed M. Hashad, Lisa M. Saksida, Tim J. Bussey

**Affiliations:** ^1^Graduate Program in Neuroscience, Western University, London, ON, Canada; ^2^Robarts Research Institute, Western University, London, ON, Canada; ^3^Department of Physiology and Pharmacology, Western University, London, ON, Canada; ^4^BrainsCAN, Western University, London, ON, Canada; ^5^Brain and Mind Institute, Western University, London, ON, Canada

**Keywords:** working memory, automated touchscreen testing, prefrontal cortex, NMDA receptors, methods for assessing rodent cognition

## Abstract

Working memory is a fundamental cognitive process for decision-making and is a hallmark impairment in a variety of neuropsychiatric and neurodegenerative diseases. Spatial working memory paradigms are a valuable tool to assess these processes in rodents and dissect the neurobiology underlying working memory. The trial unique non-match to location (TUNL) task is an automated touchscreen paradigm used to study spatial working memory and pattern separation processes in rodents. Here, animals must remember the spatial location of a stimulus presented on the screen over a delay period; and use this representation to respond to the novel location when the two are presented together. Because stimuli can be presented in a variety of spatial configurations, TUNL offers a trial-unique paradigm, which can aid in combating the development of unwanted mediating strategies. Here, we have optimized the TUNL protocol for mice to reduce training time and further reduce the potential development of mediating strategies. As a result, mice are able to accurately perform an enhanced trial-unique paradigm, where the locations of the sample and choice stimuli can be presented in any configuration on the screen during a single session. We also aimed to pharmacologically characterize this updated protocol, by assessing the roles of the medial prefrontal cortex (mPFC) and N-methyl-D-aspartate (NMDA) receptor (NMDAr) functioning during TUNL. Temporary inactivation of the medial prefrontal cortex (mPFC) was accomplished by directly infusing a mixture of GABA agonists muscimol and baclofen into the mPFC. We found that mPFC inactivation significantly impaired TUNL performance in a delay-dependent manner. In addition, mPFC inactivation significantly increased the susceptibility of mice to proactive interference. Mice were then challenged with acute systemic injections of the NMDAr antagonist ketamine, which resulted in a dose-dependent, delay-dependent working memory impairment. Together, we describe an optimized automated touchscreen task of working memory, which is dependent on the intact functioning of the mPFC and sensitive to acute NMDAr hypofunction. With the vast genetic toolbox available for modeling disease and probing neural circuit functioning in mice, the TUNL task offers a valuable paradigm to pair with these technologies to further investigate the processes underlying spatial working memory.

## Introduction

Working memory is the process by which information is held available in the absence of immediate sensory stimuli. This information can be maintained, updated, and used to facilitate decision-making and execute goal-directed behavior. In rodents, working memory is commonly assessed using a spatial paradigm, in which the location or orientation of relevant stimuli in relation to irrelevant stimuli is remembered over a brief delay period. In common working memory paradigms such as the T-maze or automated delayed match- or non-match-to-sample (DMS/DNMS) operant tasks, rodents must use information held in working memory to guide decision-making when presented with two possible choice options. Working memory has been shown to involve an interconnected system of cortical and subcortical regions (Floresco et al., 1999; Harvey et al., 2012; Eriksson et al., 2015; Zhang et al., 2019; Heikenfeld et al., 2020). Two brain regions that have been extensively studied in rodent spatial working memory are the hippocampus and medial prefrontal cortex (mPFC). Damage or disruption of the hippocampus is reliably associated with impairments in working memory, especially in paradigms using spatial cues and/or where the spatial distance between task-relevant stimuli can be made more similar (to study *pattern separation*) (Gilbert and Kesner, 2006; Kesner, 2007; Yoon et al., 2008; Clelland et al., 2009). Alternatively, the mPFC appears to be more specifically involved in performing spatial tests that require task information to be *held over a brief delay* before making a choice (Kesner et al., 1996; Lee and Kesner, 2003; Yoon et al., 2008; McAllister et al., 2013). While independent disruption of the hippocampus or mPFC reliably induces spatial working memory deficits, specific interruption of hippocampus-mPFC connections also impairs task performance (Wang and Cai, 2006; Spellman et al., 2015). Communication across the mPFC-hippocampus circuit during spatial working memory is also reflected at the cellular level via increased oscillatory synchrony between the regions during task performance (Jones and Wilson, 2005; O’Neill et al., 2013; Spellman et al., 2015; Abbas et al., 2018). As working memory deficits are a central phenotype in a variety of neurodegenerative and neuropsychiatric diseases (Glahn et al., 2005; Wang et al., 2006; Godsil et al., 2013; Schobel et al., 2013; Sigurdsson and Duvarci, 2016), established spatial working memory paradigms are a critical tool for assessing preclinical models and screening potential therapeutic agents. A critical step in the development of these tools is neurocognitive validation, which identifies the involvement of brain regions in performing a cognitive task. As such, examining the involvement of the hippocampus and mPFC during spatial working memory paradigms is important for identifying the utility of the paradigm. Therefore, establishing a high-throughput spatial working memory paradigm that can evaluate mPFC and hippocampal functioning, both memory over a delay in addition to pattern separation processes, will be an extremely valuable tool for preclinical research.

Automated paradigms provide numerous advantages toward achieving this goal, including enhanced standardization across testing sites, potential for higher throughput testing, and reduced experimenter influence (Bussey et al., 2008). However, a persistent challenge in automated rodent spatial memory paradigms has been the development of mediating strategies. Mediating strategies are behavioral processes and patterns that influence the outcome of the task but are not originally intended to be measured (Pontecorvo et al., 1996; Panlilio et al., 2011). Such behaviors have been observed in automated operant match- or non-match-to-location procedures in which rodents are required to remember the location of one of two levers over a delay and use that information to choose the correct lever when both are presented together during the choice phase (Dunnett, 1985; Dunnett et al., 1988). While such paradigms have been used extensively to assess the neural basis of working memory, the use of two consistent spatial locations throughout training and testing leaves these paradigms open to the development of alternative task-solving strategies (Pontecorvo et al., 1996). Specifically, behavioral analyses have shown that rats are able to orient their bodies toward the to-be-correct choice during the delay phase, thereby reducing or even eliminating the requirement for working memory (Chudasama and Muir, 1997; Steckler et al., 1998). Perhaps for this reason, while impairments to the mPFC consistently induced deficits in DNMTS paradigms, studies disrupting hippocampus functioning produced conflicting results (Porter et al., 2000; Winters and Dunnett, 2004; Sloan et al., 2006). Efforts to reduce mediating strategies in operant chambers have led to the development of DMTS/DNMTS paradigms that increase the number of potential response locations from a standard two-choice configuration. These include DMTS paradigms that used the 5-choice serial reaction time task operant chamber, where the number of to-be-correct locations is increased from one of two levers to one of five potential locations (Chudasama and Robbins, 2004; Chudasama et al., 2004; Teutsch and Kätzel, 2019; Kilonzo et al., 2021), and the touchscreen-based trial unique non-match to location (TUNL) task (Talpos et al., 2010; Kim et al., 2015).

The TUNL task was developed for rats by Talpos et al. (2010), who used the advantages of touchscreen-equipped operant chambers to develop an advanced spatial working memory task that was based on the widely used DNMTS tests (see Table 1). The decision to use touchscreen operant chambers was in part to address the mediating strategies that arose from a classic two-choice working memory paradigm. At the onset of a trial in the TUNL task, rats are presented with a sample light stimulus in one of fifteen locations spread across three horizontal rows (7 × 3 array) on the screen. In order to proceed, the rat must touch the sample location on the screen. Following the sample touch, the rat must move to the back of the chamber to initiate a delay period. After the delay, the rat is presented with a stimulus in the previously encountered sample location, as well as a novel location. A response to the novel location results in a reward delivery to the back of the chamber. While a single trial is still a two-choice paradigm, expanding the spatial array from two locations to several locations during training reduces the predictability of sample and choice stimuli locations (Talpos et al., 2010). Because, unlike in two-lever DNMTS, the correct location cannot be predicted, mediating responses are all but eliminated (Talpos et al., 2010). Furthermore, cognition can be probed with additional task manipulations. For example, the length of the delay period can be extended to increase the difficulty of maintaining information in working memory. Alternatively, the spatial proximity of the novel and sample location can be varied to add additional spatial demands to the task. By varying separation distance (from large to small), pattern separation processing can be assessed under increased delay conditions or independently. As a result, TUNL provides a working memory paradigm with an improved ability to assess hippocampal functioning (Gilbert et al., 1998; Gilbert and Kesner, 2006; Oomen et al., 2013; Reichelt et al., 2021). Indeed, when rats with hippocampal lesions were assessed on TUNL, choice accuracy was significantly impaired when the delay was increased, as well as when the separation distance was reduced (Talpos et al., 2010). Further characterization demonstrated that lesions to the mPFC also significantly impaired TUNL performance. Importantly, this impairment was specific to conditions in which the delay or task interference were increased, with no effect when separation distance was reduced (McAllister et al., 2013). These findings highlighted the ability to use TUNL to dissociate contributors to distinct aspects of spatial working memory. Finally, investigators have demonstrated that rats exposed to systemic N-methyl-D-aspartate (NMDA) receptor (NMDAr) antagonists, a pharmacological tool to induce neuropsychiatric-like phenotypes, demonstrate significant impairments in choice accuracy on TUNL, indicating this paradigm may be valuable for screening preclinical models (Kumar et al., 2015; Davies et al., 2017; Hurtubise et al., 2017).

**TABLE 1:** Comparison of task parameters and brain structure involvement on TUNL protocols in rats and mice.

Feature	Rat TUNL	Mouse TUNL	Mouse TUNL v2
Grid array	7 × 3	5 × 1	6 × 1

Independent training stages	Pretraining TUNL training Cognitive probes	Pretraining TUNL – stage 1 ●S3 ●S2 ●S1 TUNL – stage 2 ●S1 ●S0 TUNL – stage 3* Cognitive probes	Pretraining Cued TUNL training TUNL training ●Within session progression ●Between session progression Cognitive probes

TUNL session length	84 trials or 60 min	48 trials or 60 min	48 trials or 60 min

Delay length during training	2 s	0 s	0 s

Correction trials during training	Yes	Yes	Yes

Hippocampus involvement	°Acquisition impairment following lesion °Delay-dependent impairment with lesion °Separation distance-dependent impairment with lesion	°Re-acquisition impairment following lesion °Generalized impairment, independent of delay or separation with lesion °Sample location (center vs. non-center)-dependent impairment with lesion	°Data not available

mPFC involvement	°Delay-dependent impairment with lesion °No separation distance-dependent impairment °Interference impairment with lesion	°Data not available	°Delay-dependent impairment with reversible inactivation °Interference impairment with reversible inactivation

*Mouse TUNL v2 is the new protocol described in this paper.*

*Recently published experiments using mouse TUNL have opted out of training mice on Stage 3.

With the progression of genetic tools to develop cellular-specific neurotechnology and model the genetics of neurological disease using mice, there have been substantial efforts to develop mouse versions of touchscreen-based cognitive tests. TUNL was adapted for use in mice by Kim et al. (2015), who maintained the same task structure but reduced the number of potential locations from three rows of 21 to one row of 5 (see Table 1; But also see Josey and Brigman, 2015; Barnard et al., 2021 for multi-row mouse versions of TUNL). Mice showed similar performance patterns to rats, where choice accuracy decreased as a result of reducing the separation distance between sample and choice stimuli, as well as by increasing the delay length between sample choice stages. Importantly, mouse TUNL performance was hippocampal-dependent, though the impairment was a general reduction in choice accuracy, rather than specifically dependent on increased delay or reduced separation distance as seen in rats (Kim et al., 2015). This suggests a sensitivity of the task that may be particularly important when studying manipulations more subtle than large-scale lesions or inactivations. Further characterization demonstrated that exposure to systemic NMDAr antagonist MK-801 also significantly reduces mouse TUNL performance independent of delay length (Sokolenko et al., 2019, 2020). In the current study, we demonstrate that an updated version of the mouse TUNL protocol is also dependent on the functioning of the mPFC.

While the mouse TUNL task provides a valuable tool for studying spatial working memory and pattern separation, there are still some aspects of the paradigm that present a challenge. First, similar to the rat TUNL task, the training length is relatively extensive (Oomen et al., 2013). Of note, the original description of mouse TUNL contains a final training stage where stimuli can appear in all five windows (Kim et al., 2015 – Table 1, Stage 3), thereby substantially reducing the ability of mice to predict the correct choice. However, none of the publications since the original mouse TUNL paper have reported training mice up to this stage, likely as a result of high difficulty and required training time. An additional challenge of TUNL training is the necessity of “shaping” behavior through training, to allow animals to progress to closer separation distances. During training, mice are started at a maximum distance between sample and choice locations, and as proficiency grows across sessions, animals are trained on increasingly smaller separation distances. Importantly, these stages are isolated from each other, in that once a stage is passed, the previous trial parameters are not returned to (until later probe testing, see below). When combining this design with the one-row, five-window configuration used in mouse TUNL, it may be difficult to establish true “trial-uniqueness,” as each task stage (dictated by the separation level), effectively trains mice on a single sample and choice location. For example, at a separation distance of two windows, a sample stimulus in window 1 is always associated with a choice in window 4, and vice versa.

To improve trial uniqueness, and thus ensure avoidance of mediating responses, it would be ideal if all trial configurations, from maximum to minimum distance, were present at equal probabilities during a single session. While animals may still struggle to consistently perform minimum separation trials as proficiently as maximum separation trials, the inclusion of various trial configurations early and throughout training may act to prevent learning specific associations and the development of unwanted mediating strategies. To address this, we have adapted the TUNL protocol to maximize consistent exposure to various sample-choice configurations, while maintaining the progression of animals from maximum to minimum separation distances, throughout training. Importantly, this paradigm improves trial-uniqueness, all separation distances and trial configurations occurring at equal probabilities within a single session.

### New Protocol

**FIGURE 1:**
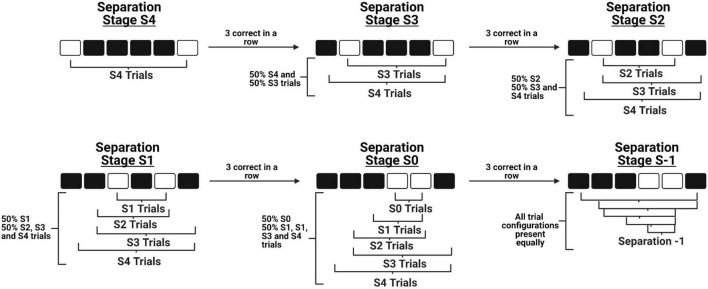
A schematic of the novel within-session progression system implemented during TUNL training. The diagram depicts how a mouse is able to progress from the maximum separation distance stage (Top left) to the minimum separation distance stage (Bottom middle) in a single session, before progressing to the full TUNL stage where all separation distances are included (Bottom right). Importantly, these stages do not contain only independent separation configurations, as previously learned configurations are still included at a lower probability across progression.

**FIGURE 2:**
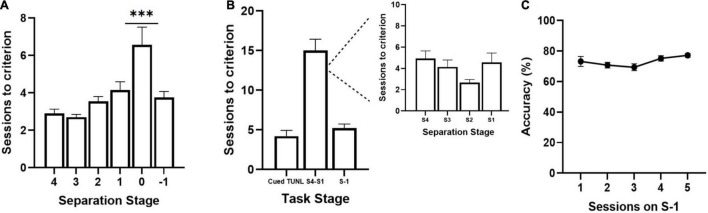
Sessions to reach criterion on TUNL stages from cohort 1 **(A)** and cohort 2 **(B,C)** of adult C57BL6 mice. **(A)** Sessions per separation stage from S4 to S-1, when mice are required to meet criterion on separation stage S0 (*n* = 20). **(B)** Session per training stage including Cued TUNL training in which mice are not required to meet criterion on separation stage S0 once they pass separation stage S1 (*n* = 9). A breakdown of sessions to criterion per separation stage is presented in the inset to the right. **(C)** Correct choice accuracy across five training sessions on separation stage S-1 (*n* = 9). Data are represented as mean ± standard error of the mean (SEM). ***p < 0.001.

There are four primary modifications we have made to the original mouse TUNL protocol (Kim et al., 2015). First, we have increased the number of windows from five to six, which allows for a greater number of configurations to be presented while maintaining that stimuli are presented along a single row. Second, we have added an intermediate stage to bridge the transition from learning to correctly touch a stimulus on the screen (“punish incorrect”) to the normal TUNL task. This stage contains the primary components of TUNL, including the sample touch, delay initiation, and choice phase. The modification is that during the choice phase, the brightness of the sample location is reduced to make it easier for mice to discriminate the correct location and acquire the non-match rule. Third, we have designed an automated *within session progression* system that allows mice to experience progressively closer separation levels within a single session as their performance improves (see Figure 1). As a result, we are able to categorize how well the mice are performing various separation distances within single sessions, as well as across training sessions. Fourth, we have characterized an alternative paradigm for running increased delay probes, which involves delivering a small amount of reward at 2 s intervals during the delay period. The objective of this alteration was to keep the mice at the back of the chamber during the entire delay period, in efforts to reduce mediating behaviors such as the animals’ tendency to return to the screen and “search” for the stimuli immediately after they have initiated the delay period (Figure 2A).

## Materials and Methods

### Apparatus

All mice were tested using standard Bussey-Saksida mouse touchscreen chambers (Campden Instruments Ltd., Loughborough, United Kingdom) as described in detail elsewhere (Horner et al., 2013; Oomen et al., 2013). The touchscreen was covered with a black plexiglass mask with two rows of six response windows, though only the six windows in the bottom row were used. The task schedules were designed, managed, and events recorded using Whisker Server and ABETTII software (Campden Instruments). A custom analysis was created using R to analyze separation level-specific performance through training.

### Task Training

#### Pretraining

Touchscreen pre-training stages were conducted as described in Kim et al. (2015). Briefly, mice were trained to obtain reward (Strawberry milkshake, Nielson) by touching a light stimulus displayed in one of the six response windows. Next, mice learned to initiate trials via nose-poking the reward tray and associate an incorrect screen response with a 5 s time-point period.

#### Cued Trial Unique Non-match to Location Training

The first stage of TUNL training is designed to teach the non-matching rule, which requires a mouse to identify the novel location as the correct choice. The sample phase begins with one of six locations being illuminated on the touchscreen. Following a nose poke to this location, the animals are directed to the back of the chamber via illumination of the reward tray [800 millisecond (ms) pulse equal to 20 μL of milkshake] and an auditory tone. If an incorrect response is made to the original sample location, a correction trial loop is initiated until the mouse makes the correct response. Correctional trials are repeated presentations of the same sample and choice locations following an incorrect response. The delay length is maintained at 0s through training and can be increased during specific probe trials. Once the back IR beams are broken following the delay period, the original sample and a novel correct location are presented simultaneously on the touchscreen. The original sample location (incorrect) is presented with 50% reduced luminance, which was implemented to aid in directing mice toward the correct location and developing the non-match rule. Mice always start the session at the maximum separation level stage, and the maximum session length is set to 60 min with a maximum trial count set to 48.

##### Within-Session Progression

Mice start training at the maximum distance between sample and correct locations (Separation distance 4 – S4, see Figure 1). Once three correct trials are completed in a row, separation distance 3 (S3) configurations are automatically integrated into the task. This initiates the animal progressing to separation stage S3, where 50% of the configurations are S4 trials and 50% are S3 trials. Again, once three correct trials are completed in a row, separation distance 2 (S2) configurations are integrated and the animal progresses to separation stage S2. Importantly, S2 configurations now constitute 50% of the trials presented, while the remaining 50% of trials are a combination of S4 and S3 trials. This design ensures that the majority of trials are separation distances that match the current stage, while also allowing animals to continue to experience configurations of previous separation distances. This progression continues through separation stages 1 and 0 (S1 and S0, respectively), until the animals reach separation stage S-1 which includes all separation distances presented at an equal probability.

##### Criterion

Mice are trained on Cued TUNL Training until they reach two consecutive sessions of completing 48 trials with an overall choice accuracy > 70%, while simultaneously reaching separation level S-1.

#### Trial Unique Non-match to Location Training

At this stage the correct choice location is no longer presented as brighter than the sample location during the choice phase. A session is finished when the mice complete 48 trials or 60 min have elapsed. During training, mice also receive a reduced reward (200 ms pulse) presented pseudorandom following sample touch on 1/3 of the trials, to encourage responding to the sample stimulus. After training is complete, and during cognitive probes and drug manipulations, sample touches are no longer rewarded.

##### Within-Session Progression

The within-session progression is the same as the previous stage, where mice begin training at the maximum separation (S4) and progress through separation levels until the reach separation stage S-1, where all separation levels occur at equal probabilities.

##### Across-Session Progression

Unlike Cued TUNL Training, mouse performance on each individual separation stage is assessed to generate across-session progression. Choice accuracy was analyzed after every testing session to determine how far the animal progressed through separation stages that day, and to determine the accuracy breakdown of each specific separation stage the animal performed. If a mouse reaches > 70% choice accuracy for two consecutive sessions on separation stage S4, on the following training session they are started at S3. Importantly, this doesn’t mean mice never see separation distance four configurations again. Similar to Cued TUNL Training, the current separation stage configurations, for example S1, are presented 50% of the time, while the remaining 50% of trial would include S4, S3, and S2 configurations. This progression continues until animals reach criterion on all separation levels. Next, animals are moved onto separation stage S-1, which represents the full TUNL task.

##### Criterion

Mice are trained on this stage until they reach two consecutive sessions of > 70% choice accuracy when started at separation stage S-1.

**FIGURE 3:**
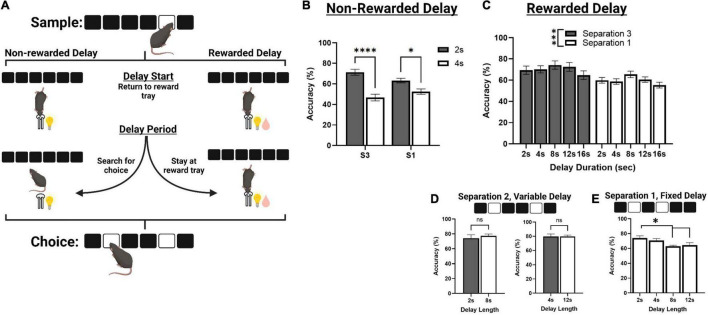
Correct choice accuracy performance across different delay protocols. **(A)** An illustration of mouse behavior during the delay phase of TUNL, representing a standard delay protocol (Left) and the novel rewarded delay protocol (Right). **(B)** Effects of delay length on correct choice accuracy during two separate mixed separation (S3 and S1), fixed delay probes (2 or 4 s) using the standard non-rewarded delay protocol. **(C)** Effects of delay length on correct choice accuracy across multiple mixed separation, fixed delay probes using the rewarded delay protocol. **(D)** Effects of delay length on correct choice accuracy during two separation mixed delay (2 and 8 s; 4 and 12 s), fixed separation (S2) probes using the rewarded delay protocol. **(E)** Effects of delay length on correct choice accuracy during multiple fixed separation (S1), fixed delay probes using the rewarded delay protocol. ****p < 0.0001, ***p < 0.001, *p < 0.05, ns = not significant (*n* = 15).

Note: It may be up to the discretion of the researcher if they are interested in having animals pass separation stage S0. S0 configurations are the most challenging as sample and correct locations are presented adjacent to each other, and therefore may add on training time for mice to reach consistently high accuracy (see Figure 3A). However, the inclusion of S0 configurations throughout training is an important aspect of the protocol in minimizing the development of mediating strategies, due to the increased unpredictability of the correct location. Furthermore, the S0 condition may be of use to those interested in evaluating hippocampal function and pattern separation, especially in experiments designed to test for performance enhancements.

#### Neurocognitive Validation

**FIGURE 4:**
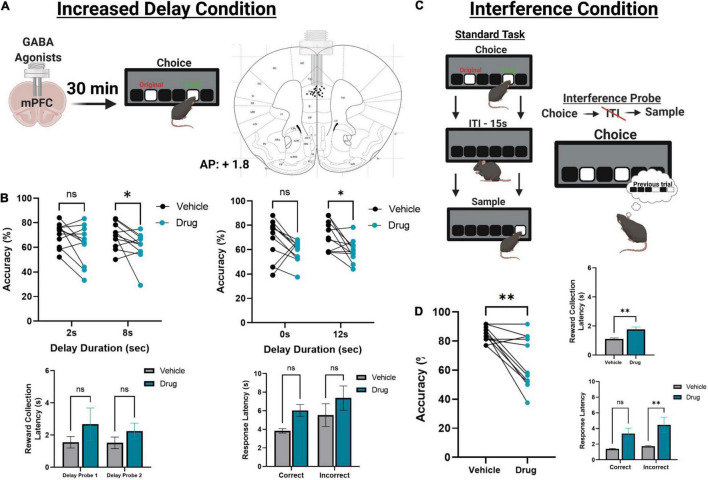
The effect of pharmacologically inactivating the mPFC on TUNL probes. **(A)** Illustration of drug infusions into the mPFC 30 min prior to testing on TUNL (left) and a drawing representation of cannula locations in the mPFC (right). **(B)** Effects of mPFC inactivation (drug) or saline (vehicle) on choice accuracy (Top left and right) and latencies (Bottom left and right) during two sessions of mixed delay, fixed separation (S2) probes. mPFC inactivation impaired choice accuracy at the high delay conditions in each probe but did not significantly alter any latency measures. Delay probe 1 refers to the mixed 2 and 8 s delays, and delay probe 2 refers to the mixed 0 and 12 s delays. Response latencies are displayed as an average across both probes for clarity. **(C)** Illustration of the interference condition, where the ITI is set to 0 s and the separation stage is S-1. **(D)** Effects of mPFC inactivation or saline on choice accuracy and latencies on the interference condition probe. mPFC inactivation significantly reduced choice accuracy, as well as significantly increased reward collection latency and incorrect choice latency. **p < 0.01, *p < 0.05, ns = not significant (*n* = 11).

We aimed to investigate whether the updated mouse TUNL task is dependent on the functioning of the mPFC, and whether acute systemic NMDAr antagonism would impair performance. Because the mPFC has been shown to be necessary for working memory across a delay and protection against interference, but not pattern separation, our probes were specific to manipulating the delay length and increasing proactive interference from previous trials (McAllister et al., 2013). We first conducted a series of probe tests to assess how increasing the delay length affected choice accuracy under various parameters with no drug manipulations. These included sessions in which: (a) the separation level was mixed between two distances and the delay was fixed at a constant length, (b) the separation level was fixed at a constant distance and two delay lengths were mixed, and (c) the separation level and delay length were both fixed for the entire session (see Figures 3B–E). To assess the effects of acute ketamine and inactivating the mPFC on working memory, we used probes with a fixed separation level (S2) and two mixed delay lengths. Further, to test the effects of acute ketamine and inactivating the mPFC on the susceptibility of working memory to proactive interference, we tested mice on separation stage S-1 with a 0 s delay but removed the 15 s ITI so the trial could occur immediately after a choice was made (see Figure 4C).

### Animals

Three cohorts of adult male C57BL6/J mice were used for this study. Two cohorts of mice (Cohort 1: *n* = 20 and Cohort 2: *n* = 9) underwent TUNL training to characterize task performance. A third cohort (*n* = 15) underwent TUNL training followed by mPFC cannulation for mPFC inactivation and systemic ketamine experiments. Mice were 9 weeks old at the start of the behavioral procedures. Mice were group-housed (2–4 animals per cage) and maintained on a 12 h/12 h reverse light cycle. All experiments were performed during the dark cycle. One week prior to training, mice were handled and habituated to the experimenter, and food was restricted to maintain 90% of free feeding body weight with *ad libitum* water throughout the experiment. Mice were habituated to the strawberry milkshake reward for a minimum of 3 days prior to training. All experiments were conducted in compliance with the standards set by the Canadian Council of Animal Care.

### Surgery

Animals were maintained under anesthesia with isoflurane and mounted in a stereotaxic frame [David Kopf Instruments. Subcutaneous Metacam (5 mg/kg) was administered prior to anesthetic induction for pain control]. Bilateral guide cannulas (PlasticsOne) were implanted to target the prelimbic region of the mPFC (AP + 1.8 mm, ML 0.3 mm from bregma, DV 2.2 mm from the skull) and secured using dental cement (Ketac Cem, 3M). Dummy cannulas were then inserted to prevent debris from entering the cannulas and covered with a plastic cap. Post-surgery Metacam was continued for 72 h for postoperative pain control, and animals were given a minimum of 7 days to recover from surgery prior to re-baseline training.

### Microinfusion Procedure and Drugs

Infusions into the mPFC were delivered through a 33-gauge bilateral injector that extended 1 mm past the end of the guide cannulas. Mice were gently restrained to insert the injector and allowed to freely move during the infusion process. The injectors were attached to a polyethylene tube connected to a 10 μL Hamilton syringe. An automated microinfusion pump (Harvard Apparatus, Holliston, MA, United States) was used to infuse 0.2 μL of a mixture of muscimol and baclofen (0.1 μg/0.1 μg) or 0.9% saline at a rate of 0.2 μL/min 30 min prior to behavioral procedures. Injectors were left in the guide cannulas for an additional one minute to allow for diffusion. Mice were habituated to the infusion process by inserting shorter injectors attached to empty syringes for three days prior to drug testing. Each animal received one drug and one saline infusion per behavioral probe, and the order of infusions was counterbalanced amongst animals. For acute ketamine exposure, mice were habituated to the intraperitoneal (i.p.) injections by inserting an empty needle tip for three days prior drug testing. Mice were injected with either 0.9% saline, 50 mg/kg, or 100 mg/kg ketamine in a random counterbalanced design. These doses have been shown to impair mouse working memory performance in the Y-maze (Hou et al., 2013; Qin et al., 2021) and have been associated with increased motor activity (Chatterjee et al., 2011; Hou et al., 2013; Qin et al., 2021). Mice were placed into the touchscreen chamber 30 min after the injection. After each pharmacological manipulation (exposure to drug or vehicle), mice were baselined on separation level S-1 with a 0 s delay for two sessions.

### Data Analysis

All data were checked by the Shapiro–Wilk test and Leven’s test for normality and homogeneity of variance, respectively. Repeated measures ANOVA were used to assess acquisition with appropriate within- and/or between-subject factors, followed by Sidak’s Multiple Comparisons *post hoc* test if there were significant main effects. Pharmacological manipulations were assessed using a two-way repeated measures ANOVA followed by *post hoc t*-tests if there was a significant result. Violation of sphericity assessed by Mauchly’s test was corrected by the Greenhouse–Geisser method. All statistical analyses were conducted using JASP.

## Results

### Trial Unique Non-match to Location Acquisition

Three cohorts of adult C57BL/6J mice were used to pilot the new version of the TUNL task. TUNL acquisition data are shown for cohorts 1 (*n* = 20) and 2 (*n* = 9) (Figure 2). Mice in cohort 1 were trained on TUNL until criterion was met for all separation levels (S4-S0), before being moved onto S-1. There was a significant main effect of separation level on sessions to criterion [*F*(2,32) = 8.17, *p* = 0.002]. *Post hoc* analysis revealed that there was a significantly higher number of sessions required to pass separation stage S0 compared to all other separation stages (*p* ≥ 0.001, see Figure 2A). Mice in cohort 2 were not required to meet criterion on separation level 0 (though S0 configurations were still present through training) and were instead moved onto separation stage –1 after passing stages 4-1 (Figures 2B,C). The average sessions to criterion on each TUNL training stage are shown in Figure 2B (Cued TUNL Training: 4.2 ± 0.74; TUNL S4-S1: 15 ± 1.5; TUNL S-1: 5.2 ± 0.54).

### Assessing the Effect of Increased Delay on Task Performance

Mice from cohort 3 were used to characterize performance on delay probes prior to surgery. First, they were assessed on standard non-rewarded mixed separation, fixed delay probes (Figures 3A,B). Here, the delay is kept constant (either 2 or 4 s) and S3 and S1 configurations are presented pseudorandomly during a single session. There was a significant main effect of delay [*F*(1,30) = 47.729, *p* < 0.001], and a significant delay × separation interaction [*F*(1,30) = 7.273, *p* = 0.011] but no significant effect of separation level [*F*(1,30) = 0.152, *p* = 0.699]. Performance was significantly reduced to chance accuracy (∼ 50%) at 4 s on both S3 and S1 trial configurations (*p* > 0.001 and *p* = 0.017, respectively, Figure 3B).

We then aimed to characterize mouse performance when a small amount of reward (80 ms pulse, compared to 800 ms for a correct choice) was delivered during the delay, in efforts to keep the animals stationary until the choice is presented (Figure 3A). Reward was delivered at 2 s intervals for delays longer than 2 s, and at the 1 s interval for 2 s delays. First, we tested mice on a series of sessions using the same fixed delay, mixed separation schedule (Figure 3C). S3 and S1 trials were presented pseudorandomly, and the delay was fixed at either 2, 4, 8, 12, or 16 s. Interestingly, there was no main effect of delay across these sessions, though it approached significance [*F*(3,77.8) = 2.712, *p* = 0.051], or delay × separation interaction [*F*(3,77.8) = 0.157, *p* = 0.924]. There was, however, a main effect of separation level on choice accuracy [*F*(1,26) = 12.169, *p* = 0.002], with mice performing significantly worse on S1 configurations compared to S3. We further assessed mouse performance on two different mixed delay (2 and 8 s; and 2 and 12 s), fixed separation level (S2) probes, though there was no significant effect of delay on choice accuracy (Figure 3D). Additionally, mice from cohort 2 were assessed on a series of fixed separation (S1), fixed delay probes (2, 4, 8, 12 s), where the delay length and separation level were constant for the entire session. Here, there was a significant effect of delay [*F*(3,24) = 5.280, *p* = 0.006], with choice accuracy being significantly reduced at 8 and 12 s compared to 2 s {[t(8) = 3.471, *p* = 0.012] and [t(8) = 2.941, *p* = 0.036], respectively. See Figure 3E}.

### Effects of Medial Prefrontal Cortex Pharmacological Inactivation on Trial Unique Non-match to Location

Following task training and surgery, mice from cohort 3 were re-baselined on separation level S-1 with a 0 s delay. Re-baselining continued until animals reached a criterion of >70% accuracy over two consecutive sessions while completing 48 trials. Additionally, mice were re-baselined for two sessions in between each pharmacological manipulation. Two mice had their data excluded due to a loss of headcap before completing all probes, and two mice were excluded due to probe placement. Otherwise, all mice were able to re-acquire the TUNL task. To test the effects of temporary mPFC inactivation during TUNL, mice from cohort 3 were assessed on two fixed separation (S2), mixed delay probes (2 and 8 s; 0 and 12 s) following an infusion of muscimol-baclofen or vehicle control (Figures 4A,B). There was a significant main effect of drug [*F*(1,20) = 7.766, *p* = 0.011], but no effect of delay [*F*(3, 60) = 0.364), *p* = 0.779]. *Post hoc* analysis of individual probe session revealed a significant reduction in performance at both 8 s [*t*(10) = 2.261, *p* = 0.047] and 12 s [*t*(10) = 2.808, *p* = 0.019]. Additionally, there were no significant effect on the latency to collect reward [*F*(1,20) = 1.277, *p* = 0.272], response latency [*F*(1,20) = 3.095, *p* = 0.094], or effect of delay on response latencies [*F*(1.9,38) = 2.530, *p* = 0.096].

Next, we aimed to assess whether increasing proactive interference from previous trials can impact performance following mPFC inactivation (Figure 4C). A significant impairment was observed following mPFC inactivation under high interference compared to controls [*t*(10) = 3.980, *p* = 0.0032]. Interestingly, on this probe there was a modest but significant increase in the latency to collect reward [*t*(10) = 3.295, *p* = 0.0093, see Figure 4D] as well as a significant effect on response latency [*F*(1,20) = 5.324, *p* = 0.032], specifically during incorrect choices [*t*(10) = 2.589, *p* = 0.027, see Figure 4D]. See Table 2 for a summary of the effects of mPFC inactivation on cognitive measures.

**TABLE 2:** Summary of the results of pharmacological manipulations on variable delay and interference probes.

Manipulation	Variable delay probes				Interference probe			
	Choice accuracy	Reward collection latency	Correct response latency	Incorrect response latency	Choice accuracy	Reward collection latency	Correct response latency	Incorrect response latency
mPFC Inactivation	Reduced (Delay-dependent)^1,2^	No effect^1,2^	No effect^1,2^	No effect^1,2^	Reduced	Increased	No effect	Increased
Ketamine – 50 mg/kg	No effect^1^	No effect^1,2^	No effect^1,2^	No effect^1,2^	No effect	No effect	No effect	No effect
Ketamine – 100 mg/kg	Reduced (Delay-dependent)^1^	No effect^1,2^	No effect^1,2^	No effect^1,2^	No effect	No effect	No effect	No effect

*Directional changes (increase or reduction) indicate significant effects on the listed measure (n = 11).*

*^1^Variable Delay Probe – 0 and 12 s.*

*^2^Variable Delay Probe – 2 and 8 s.*

### Effects of Acute Systemic Ketamine Exposure on Trial Unique Non-match to Location

**FIGURE 5:**
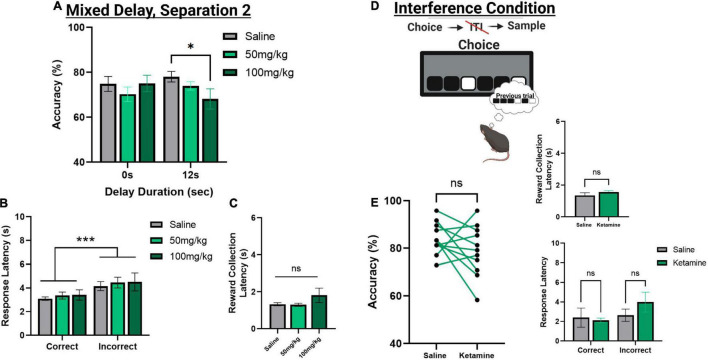
The effect of acute systemic ketamine injections on TUNL probes. **(A)** Effects of systemic ketamine doses (mg/kg) or saline (vehicle) on choice accuracy during a mixed delay, fixed separation (S2) probe. The highest ketamine dose (100 mg/kg) significantly reduced choice accuracy compared to saline at the longest delay (12 s). **(B,C)** Effect of systemic ketamine doses on response latencies **(B)** and reward correction latency **(C)**. Latency to make an incorrect choice was significantly higher than the latency for a correct choice, regardless of drug treatment. There was no significant effect of any ketamine dose on reward collection latency. **(D,E)** Effects of systemic ketamine (100 mg/kg) on choice accuracy (Left), reward collection latency (Right, top), and response latencies (Right, bottom) during the interference condition probe. Ketamine did not significantly affect correct choice accuracy or latency measures **(E)**. ***p < 0.001, *p < 0.05, ns = not significant (*n* = 11).

To assess the effects of acute ketamine exposure on working memory mice were assessed on the mixed delay (0 and 12 s), fixed separation (S2) probe. We observed a significant ketamine dose × delay interaction [*F*(2,30) = 4.561, *p* = 0.017]. *Post hoc* analysis revealed a significant reduction in choice accuracy at the 12 s delay with 100 mg/kg compared to saline [*t*(10) = 3.104, *p* = 0.011] but not when comparing 100 mg/kg to 50 mg/kg (*p* = 0.226) or 50 m/kg to saline (*p* = 0.062) (Figure 5A). When looking at choice latency, there was a significant main effect of response type [*F*(1,30) = 28.918, *p* ≤ 0.001], but no effect of drug [*F*(2,30) = 0.194, *p* = 0.824] or an interaction (Figure 5B). There was no effect of drug on the latency to collect reward [*F*(2,20) = 1.903, *p* = 0.175, see Figure 5C]. Lastly, we assessed whether the 100 mg/kg dose could affect performance on the interference probe (Figure 5D). There was no significant effect of ketamine injection on choice accuracy or latency measures compared to saline [*t*(10) = 1.359, *p* = 0.2040, see Figure 5E]. Further, neither dose of ketamine impacted overall trial count, as all mice were able to complete 48 trials within 60 min. See Table 2 for a summary of the effect of systemic ketamine on cognitive measures.

## Discussion

### Initial Task Validation

In these experiments, we set out to design an optimized version of the TUNL protocol for mice that would improve the efficiency of training while reducing the likelihood of mediating strategies (Herremans et al., 1996; Chudasama and Muir, 1997). To this end, we modified the existing TUNL protocol with a few key changes: (1) An increased spatial grid with a 6th location to add more unique trial configurations, (2) A within-session progression through task conditions that incorporates previously trained configurations with non-trained configurations, and (3) An optimized procedure for delay probes to further reduce mediating strategies and increase accuracy. To test the validity of this new mouse TUNL protocol, we attempted to replicate two classical findings in the spatial working memory literature, the involvement of the mPFC and NMDArs.

The original TUNL protocol for rats used a spatial grid consistent of seven columns and three rows (Talpos et al., 2010). The 7 × 3 spatial grid provided a reasonably large bank of trial configurations to reduce the likelihood of mediating strategies to guide responses. When the TUNL protocol was initially adapted for mice, the spatial array was reduced to a 5 × 1 grid, due to the mice having difficulty accessing additional rows (Kim et al., 2015). The loss of locations significantly impacted the number of total configurations. In the present study, we added an additional location to create a 6 × 1 grid. This was done to increase the number of spatial configurations without significantly changing the difficulty of accessing locations on the screen. While we chose to use a single row, others have also successfully designed TUNL protocols for mice utilizing multiple rows (Josey and Brigman, 2015; Barnard et al., 2021). This version of the task has been used to assess a GluN2B deletion mouse model, which revealed a generalized impairment in task performance (Josey and Brigman, 2015).

Our initial experiments demonstrated that the modifications to the training protocol resulted in a significantly shorter training period compared to the original mouse TUNL protocol (Kim et al., 2015). Indeed, mice were able to learn all but the most difficult separation level in just a few sessions. On average, mice required only 15 sessions to reach criteria on the principal task following pretraining, with an additional 5–10 sessions to acquire the most difficult spatial separation level (separation stage S0). In comparison, training with the original TUNL protocol requires multiple individual stages that take several sessions each (Kim et al., 2015). A significant decrease in training time provides enhanced utility for the TUNL task. This feature may be particularly useful when combining complex neurotechnology with the TUNL task, as these techniques can involve the implantation of sensitive probes and headcaps that can become compromised over extended periods of time. Further, mice were readily able to acquire a full version of the TUNL task, where all location windows and separation distances are included in a session at equal probabilities (separation stage –1), which may further aid in preventing mediating strategies.

The original TUNL protocol divided the training sequence into a series of isolated stages to give animals distinct exposures to all the trial configurations (Kim et al., 2015). Separation of the spatial stages was done in order to maximize the number of trials for each configuration. Isolation of trial configurations can be helpful to increase the specificity of training, but it can create additional problems. Previously, it has been demonstrated that animals completing operant tasks can become “overtrained” (Uhl, 1964; Tombaugh, 1967; Killcross and Coutureau, 2003; Pajser et al., 2021). Overtraining can lead to the development of procedural memories, by which the task is represented as a simple stimulus-response pairing. Procedural learning can create significant challenges for working memory tests, as it becomes difficult to determine if the task is measuring the short-term retention of the trial configuration, or the retrieval of a stimulus-response procedural memory (Castner et al., 2004). To address these concerns, the new TUNL protocol uses an intermixed trial presentation system during training. In training sessions, mice are given a mix of 50% trials from the trained pattern separation, while the remaining 50% are repetitions of previously trained configurations. An intermixed design reduces the risk of overtraining by ensuring that sessions have a sufficiently large number of configurations so that procedural memory is not an advantageous strategy.

Finally, we attempted to address issues with the structure of delay probe trials. In the initial characterization of mouse TUNL, the hippocampus was chemically lesioned to replicate classical spatial working memory findings in rodents (Kim et al., 2015). In experiments with limited trial configurations, mice demonstrated working memory performance up to approximately 6 s. Furthermore, lesions to the hippocampus were found to impair performance at all delays, including a 0 s delay. This result was found to be inconsistent with data from rats with hippocampal lesions performing TUNL, as lesioned rats displayed a delay-dependent impairment in accuracy at a 6-s delay, but not on a 1-s delay (Talpos et al., 2010). In order to address issues with the delay period in the mouse task, we developed several versions of the delay probe to determine the optimal parameters for assessing working memory. Initially, we started with a delay period that was either 2 or 4 s long. To prevent mice from waiting by the screen after the sample touch, the animals had to initiate the delay as well as end it by moving to the back of the chamber. In this version, we found performance on delays dropped toward chance level by 4 s, regardless of the spatial separation level. Following this, we developed a version of the delay probe where mice receive small micro-rewards (1/5th of a standard reward delivery upon correct response) at 2 s intervals during the delay (except for 2 s delay, where the interval was reduced to 1 s), to encourage mice to remain in the reward magazine for the entire duration of the delay. In this “rewarded” delay probe, we observed that mice were able to perform delays of up to 16 s with inter-mixed S3 and S1 configurations without a significant reduction in performance. This was quite unexpected, as previous studies with TUNL suggest that mice begin to show decreases in working memory performance within 2–4 s under standard conditions. We suggest the improvement in performance is due to the restriction of the animal to the reward magazine, which significantly reduces the exposure to interfering information (e.g., the spatial array at the front of the chamber). Such interference may occur as mice attempt to interact with predicted choice locations on the screen and thereby incorrectly alter the working memory representation of the original sample location. Additionally, we observed significantly lower performance when comparing S1 trials with S3 trials, independent of delay. Lower performance on smaller separation distances is in agreement with previous rat and mouse versions of TUNL, and indicates this paradigm would be valuable to assess pattern separation processes in mice (Talpos et al., 2010; Kim et al., 2015).

Finally, we were interested in whether delay probe sessions could be conducted with either variable intermixed delays, or singular fixed delays using the rewarded delay protocol. When delay probes were conducted in a variable design with S2 configurations, we were unable to detect differences between performance at a 2 and 8 s delay, or a 4 and 12 s delay. When these delays were run as fixed sessions with S1 configurations, we were able to observe a gradual reduction in performance across the delays. The variable delay probes may be more suitable for identifying deficits when the experiment is using a short-term manipulation such as a pharmacological or optogenetic methodology. Alternatively, characterization of a mouse model may be better assessed with fixed probes, as deficits can be observed more robustly at shorter delays. While we elected to use a moderate separation level (S2) for pharmacological experiments, increasing delay with smaller separation distances (e.g., S1), increases general task difficulty at baseline and may be useful for assessing more subtle manipulations.

### Inactivation of the Medial Prefrontal Cortex in Trial Unique Non-match to Location

Once we established a protocol design that effectively measured decision making in a working memory task, we tested the effects of manipulations that affected performance on previous versions of TUNL. For example, it has been reported that rats with chemical lesions of the mPFC display a selective delay-dependent impairment on TUNL at a 6-s delay, while displaying normal performance during a 0-s delay (McAllister et al., 2013). Rats with mPFC lesions were also impaired on an interference probe, where the ITI between trials was removed to increase proactive interfering information (McAllister et al., 2013). In the same study, rats with mPFC lesions showed no deficits in pattern separation using spatial separation probes. Another study took a pharmacological approach to target mPFC functioning, showing that rats infused with the NMDAr antagonist AP5 directly into the mPFC were impaired on TUNL even at a 0-s delay (Davies et al., 2017).

In order to reversibly inactivate the mPFC, mice were infused with a combination of the GABA agonists muscimol and baclofen prior to sessions. To test for working memory impairments, mice were tested on two mixed delay probes, 2 and 8 s, or 0 and 12 s. Mice treated with muscimol and baclofen demonstrated normal performance on the 0 and 2 s delay trials, while showing an impairment at 8 and 12 s. This finding replicates previous work in rats, demonstrating a delay-specific impairment in working memory (McAllister et al., 2013), as opposed to a generalized impairment, as has been reported following NMDAr antagonist infusion into the rat mPFC (Davies et al., 2017). As part of the task characterization, all mice were assessed on these delay probes prior to pharmacological manipulations. As a result, we don’t believe that the impairments seen at longer delay durations were due to novelty effects associated with a change in the paradigm. In order to determine if the mPFC is involved in the management of interfering information, mice were run on an interference probe. The interference probe was designed for TUNL following observations in the radial-arm maze paradigm, where rodent accuracy decreased within a trial as choices accumulated, indicating that interfering information from previous arm exposures may disrupt working memory (Olton, 1978; Roberts and Dale, 1981). Further, choice accuracy in the radial maze was reduced when trials were massed by reducing the ITI (Cohen et al., 1994). We observed that mice treated with muscimol and baclofen were significantly impaired on high-interference trials, which matches previously reported findings in rats (McAllister et al., 2013). Together, our results indicate that mouse TUNL is a sensitive tool for measuring mPFC function and offers conditions to assess the maintenance of information in working memory over a delay, as well as the susceptibility of held information to proactive interference.

Unlike the rat studies, we observed an increase in response latencies following medial prefrontal inactivation. There are several possible reasons why this phenomenon was observed in mice and not rats. It is possible that the administration of GABA agonists had downstream effects on motor responding that might not be observed in animals with permanent lesions, which allow greater opportunity for compensatory mechanisms to develop. This may also explain slight increases in reward collection latency observed here. Of note, all animals were still able to complete all 48 trials within an hour for each probe, regardless of drug administration. Because there is a lower motoric requirement in the touchscreens, as opposed to running in a maze, swimming, or pressing levers in an operant chamber, this paradigm may be particularly valuable for assessing spatial working memory in models of neurodegenerative diseases where motor abilities are reduced. Alternatively, slowed response latencies may reflect further dysfunction of the mPFC and indicate reductions in processing speed that slow decision-making. Interestingly, muscimol-based inactivation of the rat mPFC has been previously shown to alter response timing and reward retrieval during an operant spatial working memory task, the authors suggesting that impaired mPFC function may have disrupted the ability to integrate action-outcome contingencies over the session (Horst and Laubach, 2009). Interestingly, direct infusions of GABA_A_ antagonists such as bicuculline into the rat PFC also increases response latency in delayed working memory paradigms (Enomoto et al., 2011; Auger and Floresco, 2017). Thus, it seems that pharmacologically perturbing the mPFC excitatory-inhibitory system in either direction can alter the timing of action execution in the context of working memory in rodents.

### Systemic Administration of Ketamine in Trial Unique Non-match to Location

The importance of global NMDAr functioning for the TUNL test has been shown by previous studies in which NMDAr antagonists have been administered systemically prior to working memory assessment. Systemic administration of the NMDAr antagonist MK-801 has been shown to consistently impair choice accuracy on TUNL in rats (Kumar et al., 2015; Hurtubise et al., 2017; Lee et al., 2020). Lee et al. (2020) reported a delay-dependent decrease in choice accuracy following MK-801 administration, while Kumar et al. (2015) and Hurtubise et al. (2017) reported a general impairment independent of delay. Additionally, these studies demonstrate a general trend toward quicker responding during incorrect (Hurtubise et al., 2017; Lee et al., 2020) or correct (Kumar et al., 2015) choices. Similarly in mice, systemic MK-801 has been shown to induce delay-independent decreases in choice accuracy and response latency, as well as reduce reward collection latency (Sokolenko et al., 2019, 2020). Additionally, rats administered the NMDAr antagonist CPP were found to have a general impairment in performance on the TUNL task (Davies et al., 2017). Here, no change in reward collection or response latency was observed following drug administration. Finally, compounds that antagonize specific GluN2B-containing NMDArs, such as CP 101-606 and Ro 25-6981, have been ineffective at affecting choice accuracy on TUNL (Kumar et al., 2015; Davies et al., 2017). In our present study, we administered the NMDAr antagonist ketamine systemically into mice prior to the start of delay or interference probes. We observed that systemic ketamine resulted in a dose-dependent, delay-dependent reduction in choice accuracy, where we only observed an impairment at a 12 s delay with a high dose (100 mg/kg). No deficit was observed on the interference probe, which indicates that the potential of the interference condition to disrupt working memory performance is specific to targeted mPFC disruption. While previous studies testing NMDAr antagonists on TUNL report altered response and reward latencies, we observed no changes in latency measures, and all mice completed 48 trials within the 60 min on both probe sessions. Together, these data indicate that basic locomotor activity and goal-directed behavior were unaffected following ketamine administration.

While previous reports suggest that NMDAr antagonists like MK-801 induce generalized impairments on TUNL, our results indicate that acute ketamine exposure disrupted the maintenance of information in working memory specifically over extended delay periods. However, it is possible that a higher dose of ketamine might yield more substantial deficits at a 0-s delay. Alternatively, the differences in task design used here, compared to previous studies using MK-801 in mice (Sokolenko et al., 2019, 2020), may also contribute to these differences. In the delay probes used here, we implemented a moderate separation distance difficulty (S2), while Sokolenko et al. (2019, 2020) used a more challenging separation distance (S1). A greater requirement for pattern separation at S1 may generally increase task difficulty and result in a higher susceptibility of task performance to NMDAr antagonism at a 0-s delay. As such, these discrepancies may stem from differences in task difficulty due to the smaller spatial separations used in the studies by Sokolenko et al. (2019, 2020). Our initial observations of delay-dependent deficits following systemic administration of ketamine suggest that NMDAr dysfunction can substantially affect working memory and decision making during TUNL. As acute exposure to NMDAr antagonists has been shown to mimic cognitive impairments associated with schizophrenia, as well as exacerbate symptoms in patients (Jentsch and Roth, 1999), these findings further highlight the utility of mouse TUNL for preclinical research.

## Conclusion

The TUNL task has emerged as a valuable tool for assessing working memory and pattern separation in rodents. Variants of mouse TUNL have now been validated as sensitive to hippocampus lesions, mPFC inactivation, and NMDAr hypofunction, indicating that TUNL offers a sensitive task for studying the neurobiological mechanisms underlying spatial working memory.

Overall, the enhancements of the new TUNL protocol over existing versions provide an updated tool for researchers interested in studying decision-making in the context of working memory. Automated behavioral protocols with a shorter training period are ideal for pairing with advanced neurotechnologies such as electrophysiology, calcium imaging, chemogenetics, or mini-scopes, to further investigate the neural mechanisms underlying working memory. Further, the temporally distinct task epochs of sample, delay and choice are ideal for pairing with technologies that allow manipulations or imagining with high temporal resolution, allowing researchers to disentangle the specific mechanisms associated with specific, discrete task events. Finally, the task design of TUNL provides high face and neurocognitive validity when compared to spatial working memory tasks used to assess patients (see CANTAB), providing increased translation potential (Owen et al., 1990; Chase et al., 2008). As deficits in spatial working memory are central cognitive phenotypes in neurodegenerative and neuropsychiatric diseases, TUNL may be a valuable tool for screening prospective mouse models or assessing potential therapeutic agents.

## Significance to the Field

Deficits in working memory are foundational impairments that occur in various neurodegenerative and neuropsychiatric diseases. As working memory is a core component of functional decision-making, these cognitive impairments substantially disrupt everyday functioning. As such, spatial working memory is amongst the most readily studied cognitive processes in preclinical neuroscience research. Given the genetic advantages that mouse models provide to researchers, having a robust and well characterized spatial working memory paradigm for mice is critical for improving the translation of findings from preclinical research to clinical application. Here, we describe an optimized, completely automated, touchscreen-based spatial working memory paradigm for mice. We provide a detailed behavioral characterization and protocol of this task, which will provide an informative resource for researchers looking to study working memory on the touchscreen platform. Furthermore, we demonstrate a novel finding in that the stability of working memory information is dependent on the functioning of the mouse prefrontal cortex in this paradigm. Finally, we show that acute ketamine exposure significant impairs working memory performance when task difficulty is increased. This finding has implications for the use of this task in assessing preclinical models of diseases such as schizophrenia, as ketamine has been shown to induce schizophrenia-like cognitive impairments.

## Data Availability Statement

The raw data supporting the conclusions of this article will be made available by the authors, without undue reservation.

## Ethics Statement

The animal study was reviewed and approved by Western Animal Care Committee Affiliation, Canadian Council on Animal Care Affiliation, Ontario Animals in Research Act Affiliation, CALAM Standards of Care Affiliation, and Tri-Council Regulatory Funding Bodies (NSERC, CIHR, and SSRC).

## Author Contributions

TB, LS, and DP conceived and provided supervision for the project. DP designed and coded the behavioral task protocol and analysis programs. TD and DP designed the experiments. TD and AH performed the experiments and analyzed the data. TD, DP, and TB wrote the manuscript. All authors were involved in revising the manuscript for intellectual content, read and approved the final version of the manuscript.

## Conflict of Interest

TB and LS have established a series of targeted cognitive tests for animals, administered via touchscreen within a custom environment known as the “Bussey-Saksida touchscreen chamber.” Cambridge Enterprise, the technology transfer office of the University of Cambridge, supported commercialization of the Bussey-Saksida chamber, culminating in a license to Campden Instruments. Any financial compensation received from commercialization of the technology is fully invested in further touchscreen development and/or maintenance. The remaining authors declare that the research was conducted in the absence of any commercial or financial relationships that could be construed as a potential conflict of interest.

## Publisher’s Note

All claims expressed in this article are solely those of the authors and do not necessarily represent those of their affiliated organizations, or those of the publisher, the editors and the reviewers. Any product that may be evaluated in this article, or claim that may be made by its manufacturer, is not guaranteed or endorsed by the publisher.
